# Formation of Magnetite Nanoparticles at Low Temperature: From Superparamagnetic to Stable Single Domain Particles

**DOI:** 10.1371/journal.pone.0057070

**Published:** 2013-03-08

**Authors:** Jens Baumgartner, Luca Bertinetti, Marc Widdrat, Ann M. Hirt, Damien Faivre

**Affiliations:** 1 Department of Biomaterials, Max Planck Institute of Colloids and Interfaces, Potsdam, Germany; 2 Institute of Geophysics, ETH Zürich, Zürich, Switzerland; Massey University, New Zealand

## Abstract

The room temperature co-precipitation of ferrous and ferric iron under alkaline conditions typically yields superparamagnetic magnetite nanoparticles below a size of 20 nm. We show that at pH  =  9 this method can be tuned to grow larger particles with single stable domain magnetic (> 20–30 nm) or even multi-domain behavior (> 80 nm). The crystal growth kinetics resembles surprisingly observations of magnetite crystal formation in magnetotactic bacteria. The physicochemical parameters required for mineralization in these organisms are unknown, therefore this study provides insight into which conditions could possibly prevail in the biomineralizing vesicle compartments (magnetosomes) of these bacteria.

## Introduction

Magnetite (Fe_3_O_4_) is a naturally occurring iron oxide mineral with useful magnetic properties [1]. For this reason synthetic magnetite nanoparticles are widely studied and employed in bio- and nanotechnologies [2]. Their applications range from magnetic ink materials, ferrofluids, data recording, biomolecular scavenging to medical applications in drug delivery, magnetic resonance imaging (MRI) and hyperthermal cancer treatment [3], [4]. Interestingly, various organisms (e.g., magnetotactic bacteria, birds and fish) also produce such particles as sensors for the geomagnetic field to aid their navigation [5]. These biomineralized magnetite nanoparticles can be used by biologists and geologists as biomarkers to study current or fossil records [6], [7].

A major concern in nanoparticle synthesis is control over size. In the case of magnetite, size governs the magnetic properties [8]. Nanoparticles below a size threshold of 20 to 30 nm exhibit superparamagnetic (SP) behaviour at room temperature. Such particles do not possess a constant magnetic dipole due to thermally induced spin flipping. Above this threshold the nanoparticles are stable single-domain (SSD) ferrimagnets with an intrinsic remanent dipole. With further size increase above 80 – 100 nm, the particles magnetically divides into multiple domains (MD), lowering their magnetostatic energy [9]. Thus, particles possess the highest coercivity within the intermediate size of SSD typically ranging from 20 to 80 nm. Anisotropic particle morphologies can additionally influence these size boundaries of SSD behavior.

Multiple synthetic routes have been developed to produce magnetite nanoparticles with specified magnetic properties for given applications. These methods include aqueous co-precipitation from iron salts, sol-gel, hydrothermal, electrochemical syntheses, pyrolysis and sonolysis [1], [2], [10]. To produce particles with SSD properties or even larger MD particles, generally methods that involve elevated temperatures are required[11]. Attempts to produce nanoparticles within the SSD domain by low temperature processes in aqueous solution have failed until now [12], [13]. Therefore, it is a synthetic challenge to develop new and optimize existing procedures to obtain SSD particles under milder chemical conditions.

Remarkably, magnetotactic bacteria intracellularly form magnetite exactly in the SSD size. Thus, biology shows us that such particles can be controllably formed, at least in principle, at room temperature by mild aqueous chemistry [7]. Magnetite nanoparticles in these organisms are formed within enclosed vesicles (magnetosomes) that serve as reaction compartments for the mineralization. The process is thought to be highly controlled by the biomolecular machinery comprising many different proteins found only within the membrane of these vesicles.

Therefore, much research has been recently aimed at understanding the influence of magnetosomal proteins on magnetite crystal formation both *in vivo* [14], [15], [16] and *in vitro* [17], [18], [19], [20], [21], [22]. Specifically, the role of the protein Mms6 has been studied in much detail *in vitro* as it has been suggested to act as a nucleation and growth template for the mineral within the magnetosomes [17], [18], [19], [20], [21], [22], [23], [24]. In these synthetic assays the protein influences the crystal size: room temperature co-precipitation with protein additive leads to better constrained particle size between 20 – 30 nm, as compared to controls without the additive [17], [19], [20]. At elevated temperatures (around 90 °C) the protein restricts the size of the crystals towards smaller distributions in the partial oxidation of ferrous iron [17], [20].

Alternatively, surfactant vesicles are a much simpler approach to mimic magnetosomes as they can be tailored in the same size regime as the biological subcellular compartments [25], [26], [27]. However, magnetite precipitation within such vesicles typically leads to very small SP particles and aggregates thereof in these magnetoliposomes. This is likely caused by the high supersaturation within the vesicles before nucleation and/or the facilitated heterogenous nucleation on the membrane’s surface.

To our knowledge the possible physicochemical conditions within the magnetosomes have not been entirely considered, due to a lack of knowledge of these parameters. The resolution limits of available techniques such as pH probing with fluorescent dyes have hampered the investigation of the physicochemistry within the magnetosomes in vivo. However, it is known that magnetite formation in aqueous solution requires at least moderately alkaline (pH ≥ 8.5) and anoxic conditions at room temperature [1], [7], [28].

Thus, the closest biomimetic approach in terms of physicochemistry (i.e. temperature, pressure, solvent, pH and ionic strength) is the co-precipitation of ferrous and ferric iron salts by alkaline hydrolysis. While earlier reports envisaged a role of ferrihydrite as precursor in magnetite biomineralization in magnetotactic bacteria [29], [30], we have since shown that the observed ferritin pool in the cells was not connected to mineralization [31]. This co-precipitation method typically delivers only SP particles in the size range 2–13 nm [32], [33]. In this range size tailoring can be achieved by adjusting the parameters pH and ionic strength. Particle size decreases with increasing pH and ionic strength due to lower surface tensions of the particles in these milieus [33]. Typically, the physiological pH in bacteria is assumed neutral, but unicellular eukaryotic calcifying organisms have been shown to locally elevate the intracellular pH ≈ 9 to promote biomineralization [34]. Thus, it seems reasonable to assume that magnetotactic bacteria are able to achieve a similar pH within the magnetosome compartments to achieve mineralization of magnetite.

In this study we demonstrate that co-precipitation of ferrous and ferric iron at pH  =  9 without additives can deliver SSD and MD magnetite nanoparticles and that the growth kinetics under these conditions resemble earlier reports on magnetite growth in magnetotactic bacteria [31], [35].

## Results and Discussion

**Figure 1:**
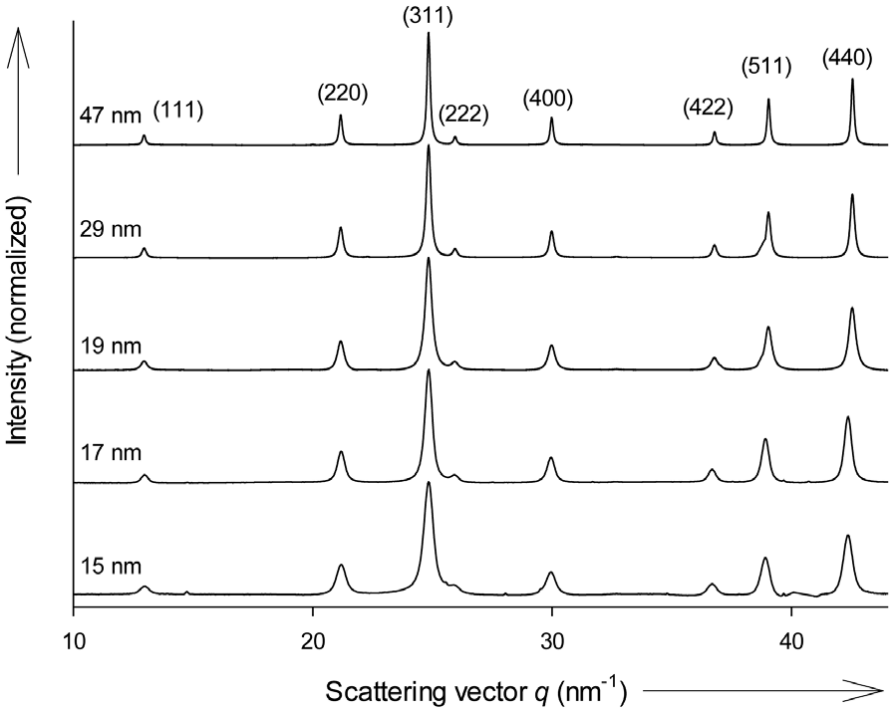
XRD patterns obtained of co-precipitation products. Increasing growth times yield larger particles as indicated by narrowing Bragg peaks. Magnetite peak indexing is given in brackets. Unindexed weak peaks are from an internal α-SiO_2_ standard for sample-detector distance calibration.

We use a titration device equipped with micro-capillary inlets, which enables ultraslow and constant solution dosing and the concomitant adjustment of pH during synthesis (Figure S1). Due to the extremely low solubility of iron in neutral and alkaline media, fast addition of iron leads to immediate supersaturation, which results in rapid nucleation of only small magnetite nanoparticles [28], [33], [36]. Growth beyond the SSD threshold is achieved at dosing rates of 1 µL min^-1^ with 56 µg Fe min^-1^ of iron chloride solutions to sodium hydroxide at pH  =  9. We used synchrotron X-ray diffraction (XRD) to characterize the phase and the average grain size of the precipitated material as given by the Bragg reflections and determined by Scherrer peak broadening analysis. Figure 1 shows diffractograms of magnetite particles with mean particle sizes ranging from 15 to 47 nm as obtained for samples after 5 to 480 minutes growth, respectively.

**Figure 2:**
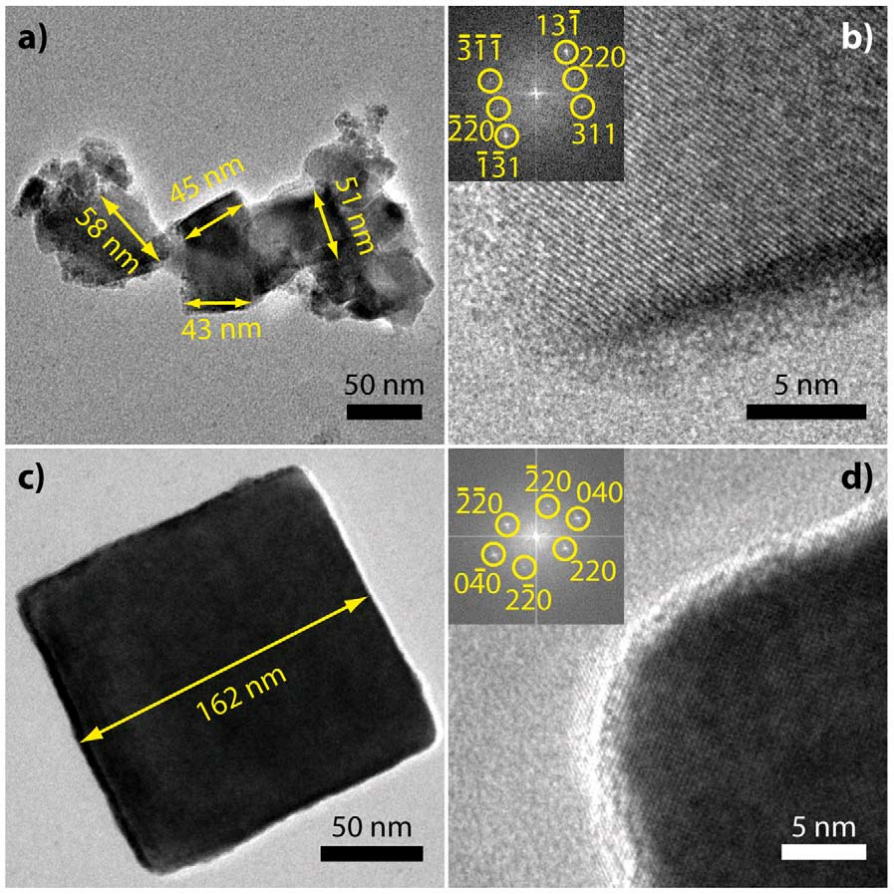
TEM images of magnetite nanoparticles grown for 480 min. **a)** Aggregate composed of several particles in the stable single-domain size range and smaller particles. **b)** HRTEM of edge of the 45 nm marked particle in (a), inset: FFT with indexed reflections, zone axis [-112]. **c)** large, presumably multi-domain particle, **d)** HRTEM of particle corner in (c), inset: FFT with indexed reflections, zone axis [001].

**Figure 3:**
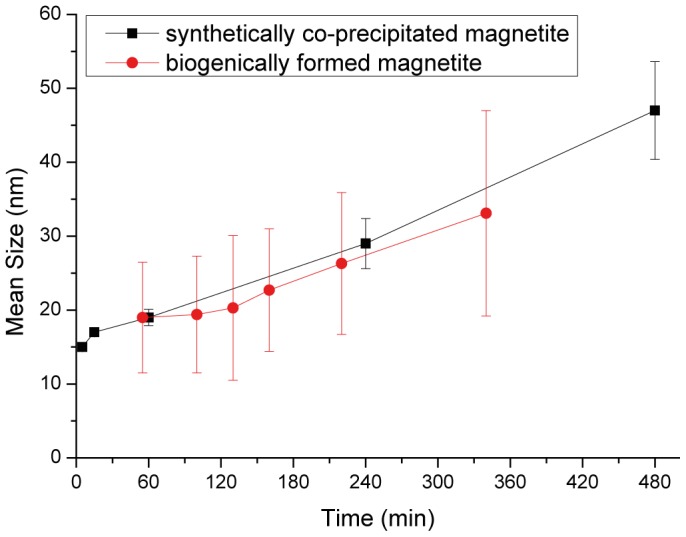
Plot of particle size over growth time for the synthetic particles (black squares, size measured from peak broadening of XRD patterns) and magnetite particle growth within magnetotactic bacteria *M. gryphiswaldense* (red circles, size measured from TEM images as described in [31], [35]). Error bars represent standard deviations of the mean particle sizes determined by XRD peak broadening for synthetic samples (black) and standard deviations of crystal size distributions measured by TEM for bacteria (red).

Both transmission electron microscopy (TEM, Figure 2) and XRD confirm that magnetite can be grown within a few hours beyond the SP / SSD threshold of 20 – 30 nm. Although the particles are obtained as poly-dispersed aggregates (Figure 2a), the average grain size increases with time as measured from from peak broadening of XRD patterns (Figure 3). The smallest particles observed in Figure 2a could not be identified as magnetite by high-resolution imaging and may be reaction intermediates In contrast to previous experimental procedure that resulted only in small SP nanoparticles, the ultraslow addition, which we use in this study, [28], [33] probably reduces or avoids the continuous nucleation in favor of growth of the nucleated material, thus enabling formation of larger particles in the SSD size range. Interestingly, these particle growth kinetics, which are observed in our system at pH  =  9, are very similar to the biomineralization of magnetite in the magnetotactic bacterial species *M. gryphiswaldense* (Figure 3)[31], [35]. The Eh-pH stability domain of magnetite starts around 8 and finishes at around 14, depending on the iron concentration [7]. Therefore, a pH of 9 is not surprising as it is at the rather lower end of this domain. In addition, a new alkaliphile magnetotactic bacterial strain was isolated last year [37], showing that at least some of these type of organisms can cope with such environmental conditions.

To evaluate the oxidation of the synthetic magnetite to maghemite, we performed high resolution X-ray diffraction measurements on a series of magnetite particles formed over a period of 1 to 100 min. The measurements reveal a decreasing lattice parameter from 0.83895 ± 0.00024 down to 0.83664 ± 0.00055 nm with decreasing particle size (Table 1). Calculating the oxidation parameter z using the formula Fe^III^(Fe^II^_(1-z)_Fe^III^_(1+2z/3)_[ ]_z/3_ to describe the solid solution between magnetite (z  =  0) and maghemtite (z  =  1), we obtain oxidations between 0.26 for the largest down to 0.79 for the smallest particles [38]. Assuming an original pristine homogeneous magnetite particle getting oxidized and resulting in a magnetite core with a surrounding maghemite layer in which the volume of the inner core is given by (1-z)V_total_, we can calculate that this observation is consistent with surface oxidation of 1 – 2 nm of the outer particle layers to maghemite.

**Table 1:** High resolution X-ray diffraction measurements, derived oxidation parameter and average oxidized layer thickness.

Growth Time (min)	Avg. Particle Size by XRD (nm) ± std error	Lattice parameter a by XRD (nm)	Oxidation parameter z Fe^III^(Fe^II^_(1-z)_Fe^III^_(1+2z/3)_[ ]_z/3_	Avg. Oxidation layer thickness (nm)
1	7.6 ± 0.2	0.83664 ± 0.00055	0.79	1.5
5	14.5 ± 0.3	0.83772 ± 0.00079	0.6	1.9
10	16.0 ± 0.3	0.83832 ± 0.00018	0.46	1.5
30	16.0 ± 0.3	0.83869 ± 0.00021	0.35	1.1
100	19.4 ± 0.4	0.83895 ± 0.00024	0.26	0.9

**Figure 4:**
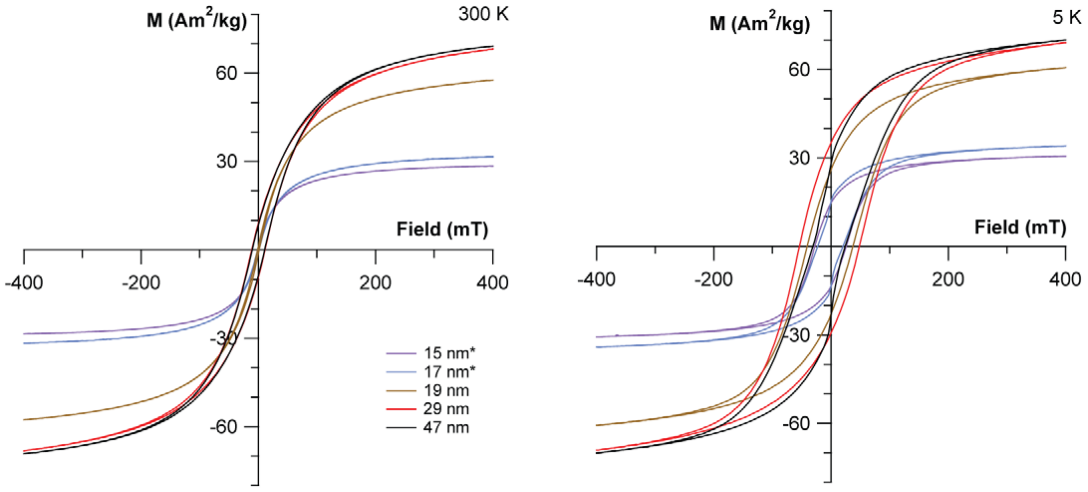
Magnetic properties at 300 K and 5K. Hysteresis measurements of magnetite with increasing particle size. Note that samples marked with (*) were estimated in µg range.

To determine whether the formed particles indeed also exhibit the expected magnetic properties, magnetic hysteresis measurements were performed on a superconducting quantum interference device (SQUID) at 300 K and 5 K in a maximum field of 400 mT. Figure 4 shows saturation-normalized hysteresis data of the precipitates at 300 K (see also Table 2).

**Table 2:** Summarized properties of the synthesized magnetite particles including the required growth time, the mean grain size as determined by XRD and coercivity at 300 K and 5 K as determined by hysteresis measurements.

Growth Time (min)	Mean Size (nm)	Coercivity *H*_C_ 300 K (mT)	Coercivity *H*_C_ 5 K (mT)	Saturation Magnetization M_S_ (A m^2^ kg^-1^)	Remanence M_R_ (A m^2^ kg^-1^)
5	15±0.3	0.3	26.9	(*)	(*)
15	17±0.3	1.2	22.5	(*)	(*)
60	19±1.1	4.5	45.0	52	3
240	29±3.4	11.5	51.3	68	9
480	47±6.6	10.6	27.7	69	8

(*) not determined due to low sample amount.

The hysteresis measurements clearly reveal a continuous increase in coercivity *H*_C_ and magnetization ratio *M*_R_/*M*_S_ with increasing grain size, reflecting the increase in particle size from SP to SSD (Figure 4a and b). While the particles of 15 nm size show practically no hysteresis, which means that most particles are superparamagnetic (*H*_C_  =  0.3 mT, *M*_R_ / *M*_S_  =  0.01), particles of 29 nm have *H*_C_  =  11.5 mT and *M*_R_/*M*_S_  =  0.13, which indicates a blocked magnetization that is typical for magnetite particles obtained through high temperature synthetic routes [11]. A slight decrease in *M_s_* and *H_c_* is observed for the largest particles (mean 47 nm). Additionally, we observe a wasp-waisted hysteresis for the largest particles at 5 K, which indicates that the material is composed of at least two distinct magnetic phases (Figure 4)[39]. This is consistent with the presence of even larger particles with multi-domain size of 100 nm as seen in electron microscopy (Figure 2c), but obscured in XRD due to instrumental broadening resolution limits for such large particles. The saturation magnetizations *M*_S_ for the ferrimagnetic stages were ∼ 68 – 69 A m^2^ kg^-1^ in good agreement with reported values for surface-oxidized magnetite nanoparticles and with the oxidation parameter determined by HRXRD [40].

Further magnetic tests were carried out to confirm the particle size and purity of magnetite particles. Purely stoichiometric magnetite undergoes a change in its electrical and magnetic properties around 120 K, known as the Verwey transition [11], [40], [41]. This Verwey transition is a change in the electron ordering between the octahedral site Fe^III^ and Fe^II^ atoms of the spinel structure, which causes a crystallographic phase transition from a cubic to a monoclinic lattice at *T*_V_ [41], [42]. It is suppressed if particles are oxidized or have very fine size [40], [43]. Magnetic bacteria often show a transition around 100 K [44]. In the first test, samples were cooled to 5 K in a 400 mT field, thus applying a saturation remanent magnetization; the change in *M_R_* was then monitored during warming from 5 K to 300 K (Figure S2). A change in slope in the warming curve at approximately 100 K, which can be attributed to the Verwey transition, is clearly seen for ferrimagnetic particles of 29 and 47 nm, and to a lesser extent for 19 nm. Finer particles do not show a transition.

Parallel to these measurements, initial susceptibility was also measured during warming, using a 318 A m^-1^ alternating field and frequencies of 1, 11 and 110 Hz. This allows extraction of the in-phase and quadrature component (not shown) of susceptibility (Figure S3). The 19 nm, 29 nm and 47 nm particles show a distinct change in curvature of the warming curve at 100 K consistent with a Verwey transition. The susceptibility of the 47 nm particles shows very little frequency dependence, which suggests that there are few SP particles; the majority of the particles are magnetically blocked. The other samples show a greater degree of frequency dependent susceptibility, which indicates a broader distribution in particles size.

The magnetic properties of the obtained synthetic particles are not as pronounced as their biogenic archetypes. Reported coercivities for magnetite nanoparticles within the magnetosomes of magnetotactic bacteria at room temperature are between 15.5 and 27.0 mT [44], [45]. However, in contrast to the synthetic particles, magnetosomes are very monodisperse, and aggregation is prevented due to their shielding phospholipid-membrane. Additionally, another major contribution to the observed differences can be attributed to the surface oxidation of the synthetic particles that we observe in every sample irrespective of their dimensions, while magnetotactic bacteria are able to preserve stoichiometric magnetite, due to the presence of the biological organelle and membranes [38].

## Conclusions

In summary, we showed that co-precipitation of ferrous and ferric iron at moderately alkaline pH  =  9 allows the formation of SSD to MD magnetite nanoparticles at ambient conditions (room temperature and atmospheric pressure). Our findings will help contribute to a better understanding of magnetite biomineralization, as the required growth times in our system are very similar to observations in magnetotactic bacteria. Further investigations, using this fabrication method, can help resolve the yet unknown physicochemical parameters for magnetite precipitation *in vivo*. We envision our titration system to enable more controlled mineralization studies with magnetotactic bacterial proteins under mild and more native conditions than previously conducted. Furthermore, we anticipate our synthesis to be the starting point towards a wider application of magnetic nanoparticles where permanent magnetic properties are needed.

## Materials and Methods

The experimental setup is a computer-controlled titration device (Metrohm AG) consisting of a dosing unit 776 Dosimat (1 mL cylinder), a titration unit 719 Titrino (5 mL cylinder) and a Biotrode pH electrode. Reactions were performed under nitrogen atmosphere in a 50 mL titration vessel with thermostat jacket, which was kept at constant temperature (25±0.1°C) with a thermostat M3 (Lauda). Iron chloride solutions (1 M) with a stoichiometric ratio Fe^II^ / Fe^III^  =  1 / 2 were prepared from ferrous chloride tetrahydrate (VWR) and ferric chloride (Sigma-Aldrich). Sodium hydroxide solutions (1 M, Merck) were used as supplied. Deionized water and all solutions were initially purged with nitrogen before use. Magnetite was precipitated by addition of the Fe solution (1 µL min^-1^) through a ∼ 200 µm wide polypropylene capillary (Microloader, Eppendorf) to the dilute NaOH (10 mL) in the reaction vessel. Before and throughout the precipitation the pH is kept at 9 ± 0.4 by addition of NaOH as controlled by the setup. The precipitates were washed with deionized water and dried. TEM images were acquired with a Zeiss LIBRA 200FE operated at 200 kV using a Gatan US1000 CCD camera at the Helmholtz Zentrum Berlin. An in-column energy filter was employed to obtain zero-loss filtered bright-field images. XRD was measured at the µ-Spot synchrotron beamline (BESSY II, Helmholtz Zentrum Berlin) with a 100 µm beam of 15 keV [46]. Nanoparticle dimensions are calculated from Scherrer peak broadening analysis of the XRD peaks obtained following a procedure earlier followed in our group where the instrumental peak broadening is taken into account [38]. Magnetic measurements were performed using a Quantum Design Magnetic Properties Measurement System (MPMS XL-7) SQUID magnetometer at the University of Bremen.

## Supporting Information

Figure S1 **Schematic drawing of the reactor used for magnetite co-precipitation.**
(TIF)

Figure S2 **Remanence as a function of temperature after field cooling at 5 K in a 400 mT field.** Inset is the derivative of the heating curve- Kinks (arrows) in the remanence and derivative curves around 100 K indicate the Verwey transition in precipitates grown for 60, 240 and 480 minutes (mean 19, 29 and 47 nm, respectively).
(TIF)

Figure S3 **AC magnetization induced with an AC field using frequencies of 1 Hz, 11 Hz and 110 Hz as a function of temperature.** Kinks (arrows) in the magnetization curve around 100 K indicate the Verwey transition in precipitates grown for 60, 240 and 480 minutes (mean 19, 29 and 47 nm, respectively). AC magnetization for the 5min and 15 min precipitates were too weak to measure.
(TIF)
