# Supplementary figures and images for: Formation of Magnetite Nanoparticles at Low Temperature: From Superparamagnetic to Stable Single Domain Particles
Source: PLoS One. 2013 Mar 8;8(3):e57070. doi: 10.1371/journal.pone.0057070 (PMC3592859; doi:10.1371/journal.pone.0057070)

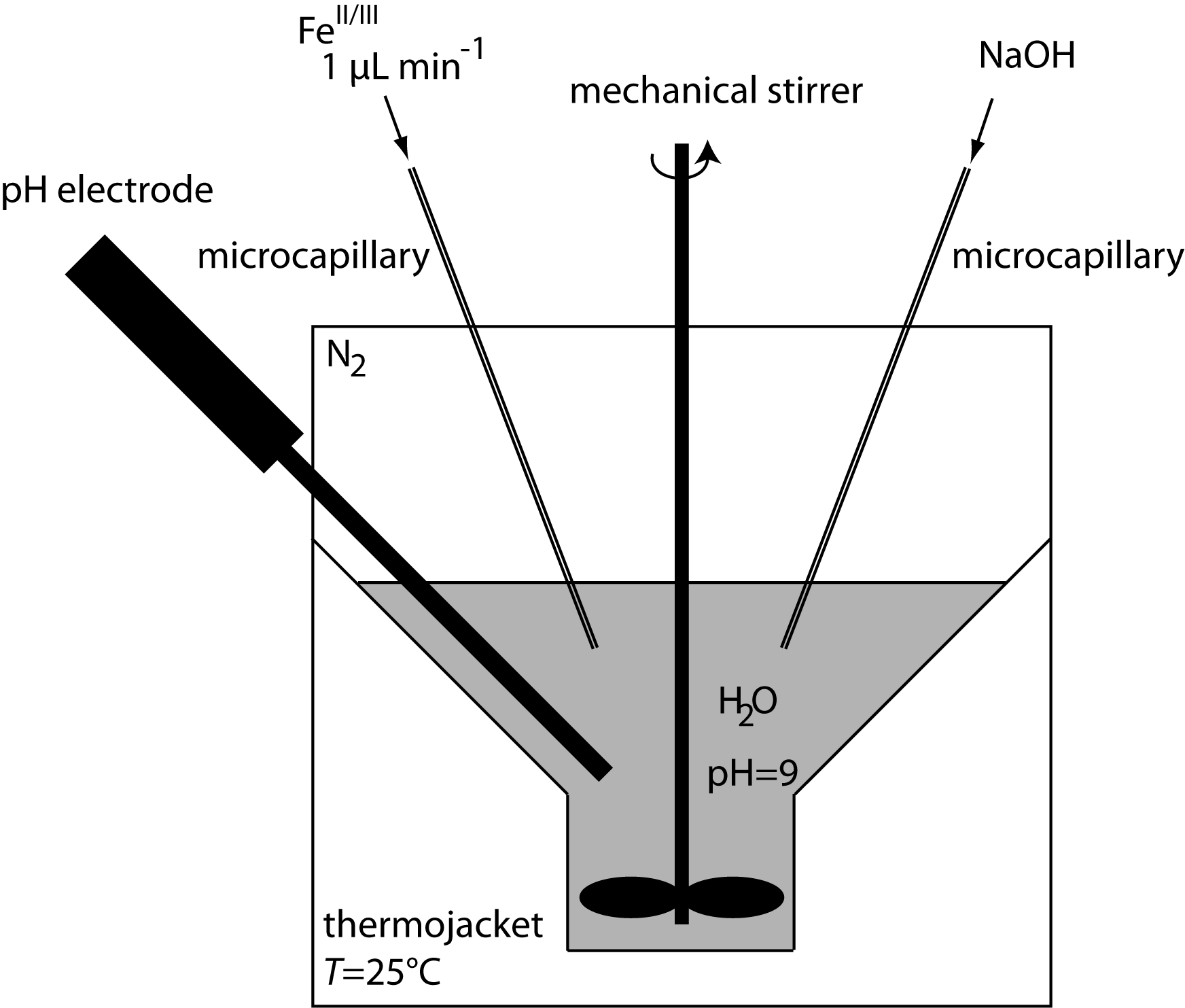

Supplement: Figure S1 — Schematic drawing of the reactor used for magnetite co-precipitation. (TIF) [file pone.0057070.s001.tif]
